# The Utility of Shallow RNA-Seq for Documenting Differential Gene Expression in Genes with High and Low Levels of Expression

**DOI:** 10.1371/journal.pone.0084160

**Published:** 2013-12-16

**Authors:** Joel Atallah, David C. Plachetzki, W. Cameron Jasper, Brian R. Johnson

**Affiliations:** 1 Department of Entomology, University of California Davis, Davis, California, United States of America; 2 Department of Ecology and Evolution, University of California Davis, Davis, California, United States of America; Schulze Center for Novel Therapeutics, Mayo Clinic, United States of America

## Abstract

The sequencing depth necessary for documenting differential gene expression using RNA-Seq has been little explored outside of model systems. In particular, the depth required to analyze large-scale patterns of differential transcription factor expression is not known. The goal of the present study is to explore the effectiveness of shallow (relatively low read depth) RNA-Seq. We focus on two tissues in the honey bee: the sting gland and the digestive tract. The sting gland is an experimentally well-understood tissue that we use to benchmark the utility of this approach. We use the digestive tract to test the results obtained with the sting gland, and to conduct RNA-Seq between tissue types. Using a list of experimentally verified genes conferring tissue-specific functions in the sting gland, we show that relatively little read depth is necessary to identify them. We argue that this result should be broadly applicable, since genes important for tissue-specific functions often have robust expression patterns, and because we obtained similar results in our analysis of the digestive tract. Furthermore, we demonstrate that the differential expression of transcription factors, which are transcribed at low levels compared to other genes, can nevertheless often be determined using shallow RNA-Seq. Overall, we find over 150 differentially expressed transcription factors in our tissues at a read depth of only 12 million. This work shows the utility of low-depth sequencing for identifying genes important for tissue-specific functions. It also verifies the often-held belief that transcription factors show low levels of expression, while demonstrating that, in spite of this, they are frequently amenable to shallow RNA-Seq. Our findings should be of benefit to researchers using RNA-Seq in many different biological systems.

## Introduction

Next-generation sequencing has greatly expanded our capacity to address fundamental questions in genomics [1-4]. RNA-Seq has allowed for great resolution in quantifying differentially expressed genes, in new gene discovery, and in documenting patterns of alternative splicing [5-10]. Protocols also allow for exploring patterns of microRNA expression and for locating the binding sites of transcription factors [11-14]. Along with increases in sequencing hardware, a multitude of different software packages are currently available for analyzing next-generation data sets [15-18]. While much work remains to be done in the analysis domain, progress has been made and important analyses are being conducted on many biological topics [19-23].

In spite of the progress of the past several years, there are still basic questions relevant to the use of RNA-Seq that remain unanswered for most organisms. Work on the sequencing depth necessary for identifying differentially expressed genes, for example, has been conducted primarily with mammals, and it is not clear that equal depth is necessary for organisms with simpler transcriptomes [18,24,25]. Second, quantifying the read depth necessary for RNA-Seq might depend on whether the focal genes show high or low levels of expression. In particular, transcription factors (TFs) are thought to be expressed at relatively low rates, but few studies have documented how low these rates are, and what sequencing depth is necessary to document differential expression in these genes [26-28].

An approach to the problem of sequencing depth that has not been used, but could be productive, is to use well-established bodies of experimental work on particular tissues to benchmark how many reads are necessary to identify key genes. The sting gland of the honey bee is such a tissue and is partly the focus of the present study. Honey bee venom, because it can cause serious allergic reactions, has been the subject of intense experimental work [29-31]. All of the major venom components of honey bee venom are known, as are many of the allergens present in venom. Further, it is known that the filling of the venom gland shows a developmental signature, as the oldest bees in a nest, the foragers, have mature venom sacs, and are likely to sting, while younger bees, nurses, have incompletely filled venom sacs and are unlikely to sting. Previous work has shown that venoms are transcribed at higher rates in young bees, but the resulting proteins do not reach high concentration in the venom sac until later in life [32,33]. At maturity, low levels of expression for venoms continue [34], but at reduced rates relative to when the venom sac is filling. Hence, many of the functional venom genes show differential expression between developmental life history phases (nurses and foragers). Venoms, and genes associated with them, are also likely differentially expressed between tissues, as these genes are specialized and not thought to be used elsewhere in the body.

The concept of shallow RNA-Seq is that important biological insights can be made with sequencing depths well below that necessary for achieving saturation with respect to the total number of genes found to be expressed, or differentially expressed [35]. One might assume that advances in sequencing technology, which make it possible to obtain hundreds of millions of reads from a single sequencing lane, would obviate the need for shallow RNA-Seq. However, there has been a concomitant increase in the number of samples which can be multiplexed on a lane. Twenty-four barcodes can be obtained from Illumina Truseq RNA kits, and a recently developed protocol provides 96 [36]. Shallow RNA-Seq allows the option of increasing the number of assayed samples without elevating the cost. Given that the major weakness of current RNA-Seq studies is low number of biological replicates, determining the optimal number of reads per sample is of vital importance. 

Our goal in this study is to show that shallow RNA-Seq can be used to find certain classes of functionally important genes. Our experimental approach is to first demonstrate that the sequencing depth we use is below saturation level. We then determine whether shallow RNA-Seq is capable of identifying as differentially expressed the genes in the sting gland conferring tissue specific functions. Having shown that these key functional genes can be identified with shallow RNA-Seq, we turn to the question of whether such genes would be recognizable as particularly important, were they not already experimentally characterized. We do this in two ways: first, we explore the expression patterns of the key genes to show that they stand out relative to other differentially expressed genes (DEGs), and second, we explore whether the identification of genes important for the tissue specific functions of the digestive tract are amenable to the same approach. Following this work on genes conferring tissue specificity, we explore whether shallow RNA-Seq can also be used to identify differentially expressed transcription factors. Transcription factors are expressed at lower rates and strongly differ in function relative to the focal genes from the previous analyses. Determining whether the same shallow RNA-Seq approach has utility for the study of these genes is therefore a good test of how broadly applicable our results are likely to be. We show that the study of TFs is also possible with shallow RNA-Seq. 

## Materials and Methods

### Colonies and collection of bees

Honey bee colonies were kept using standard beekeeping practices at the Laidlaw Honey Bee Research Facility on the UC Davis Campus. Bees (nurses and foragers) were collected from 2 full-size hives. Nurses were collected by opening the nest and identifying individuals with their heads and thoraxes inside of brood cells for at least 3 seconds [37,38]. A further confirmation of nurse bee developmental status was made at the time of dissection by cutting into the head capsule. Nurses have Hypopharyngeal glands that are much more highly developed than those of foragers and are different in color [39,40]. Glandular material spills out readily when nurse head capsules are opened, while this does not occur in foragers. Nurses without large HP glands were discarded, as were foragers without small HP glands. Pollen foragers were used for all analyses. All bees were stored at -80°C until time of dissection.

### Dissections, Extractions, and Sequencing

We used two biological replicates in this study. For a particular replicate, a pool of bees from the same colony was used. Different replicates were composed of pools of bees from different colonies. Tissues were pooled because some of the structures were small, and would not provide sufficient RNA alone. Pooling was also done to control for variance between bees, since RNA-Seq studies cannot use large sample sizes due to cost. Total RNA was extracted using Trizol according to the manufacturer’s instructions. For each library (8 total) RNA from 30 individuals was pooled. For the sting gland, each bee was individually removed from the freezer and allowed to thaw in a glass dissection dish full of 50% ethanol. As soon as the abdomen was thawed, the stinger was grasped with forceps and gently pulled from the abdomen. The honey bee stinger, gland, and venom reservoir are all designed to easily detach from the body of the bee when it stings, making this dissection simple. Each individual stinger (and associated gland) was then washed in fresh 50% EtOH and homogenized in Trizol before repeating the procedure with the next bee. For the digestive tract, a similar procedure was used except that the abdomen was cut from the rest of the body before the digestive tract was dissected out, rinsed in 50% ETOH, and homogenized in Trizol. Since the digestive tract was much larger than the sting gland, 10 1.5 ml tubes were used for extraction (3 digestive tracts per tube). After all 30 extractions were complete, all the homogenized material was placed in one 50 ml centrifuge tube and vortexed. One ml of homogenized tissue in Trizol was then pipetted into a fresh 1.5 ml tube for RNA extraction. Total RNA was quality checked with the Agilent Bioanalyzer 2100. Libraries were generated according to the Illumina TruSeq v2 kit instructions. Libraries were sequenced on an Illumina HiSeq 2500 machine. 100 bp paired-end sequencing was performed. The raw data from this study has been submitted to the NCBI SRA archive (SRP020361).

### Quality Control

Standard quality control steps (using FastQC and fastx-toolkit) were taken to remove low quality reads and base calls. First, all low quality reads were removed (reads with greater than 33% Ns or average quality < 25 phred score). Then each read was assessed with a sliding window from the right end such that the average quality score at that end was > 25. Finally, reads were searched for Illumina adapter contamination using the Cutadapt program [41]. All libraries had overall mapping rates to the honey bee genome greater than 80%.

### RNA-Seq analyses

To generate data sets of different sequencing depth, reads were sampled from much larger libraries at random. Hence, for the 1 million read analyses, one million reads from each replicate were randomly sampled from a much larger sequencing run. Reads were aligned to the most recent public release of the honey bee genome (4.0). Analyses were conducted with two software packages: DESeq, and NOISeq [16,18]. In each case, Tophat v2.0.4 was used for alignment with bowtie 2.0 [15]. We used the HT-Seq package for determination of reads mapped to each gene. For NOISeq, we ran the analyses with both RPKM and upper quartile normalization procedures, and used p = 0.8 as our cutoff for statistical significance (the developers’ recommended value). For DESeq we used an adjusted p value of < 0.05 as the criterion for differential expression. 

### Quantitative real-time PCR

Real-time PCR validation was carried out on a Biorad CFX96 RT-PCR detection system, following established protocols, for 10 genes found to differ between nurses and foragers in the RNAseq digestive tract analysis. Primers are listed in Table S1. Calibration curves were generated to determine the efficiency of the reaction at each temperature. RNA extracted from three biological replicates (each from a separate colony) of nurse and forager digestive tracts was used. The dissections followed the same protocol that was utilized for the RNA-seq samples, but the source material was from different colonies. Reverse transcription was carried out separately on three technical replicates of each biological replicate, using the Biorad iScript reverse transcription supermix. Each technical replicate was then used in the qPCR assays. The results were analyzed using the Bio-rad CFX Manager 3.0 software and the R programming language. The honeybee homolog of *ribosomal* protein *49* (*RP49*) was used as a reference gene. This gene was previously validated for qPCR normalization in honeybees [42].

## Results and Discussion

### Total numbers of differentially expressed genes

**Figure 1:**
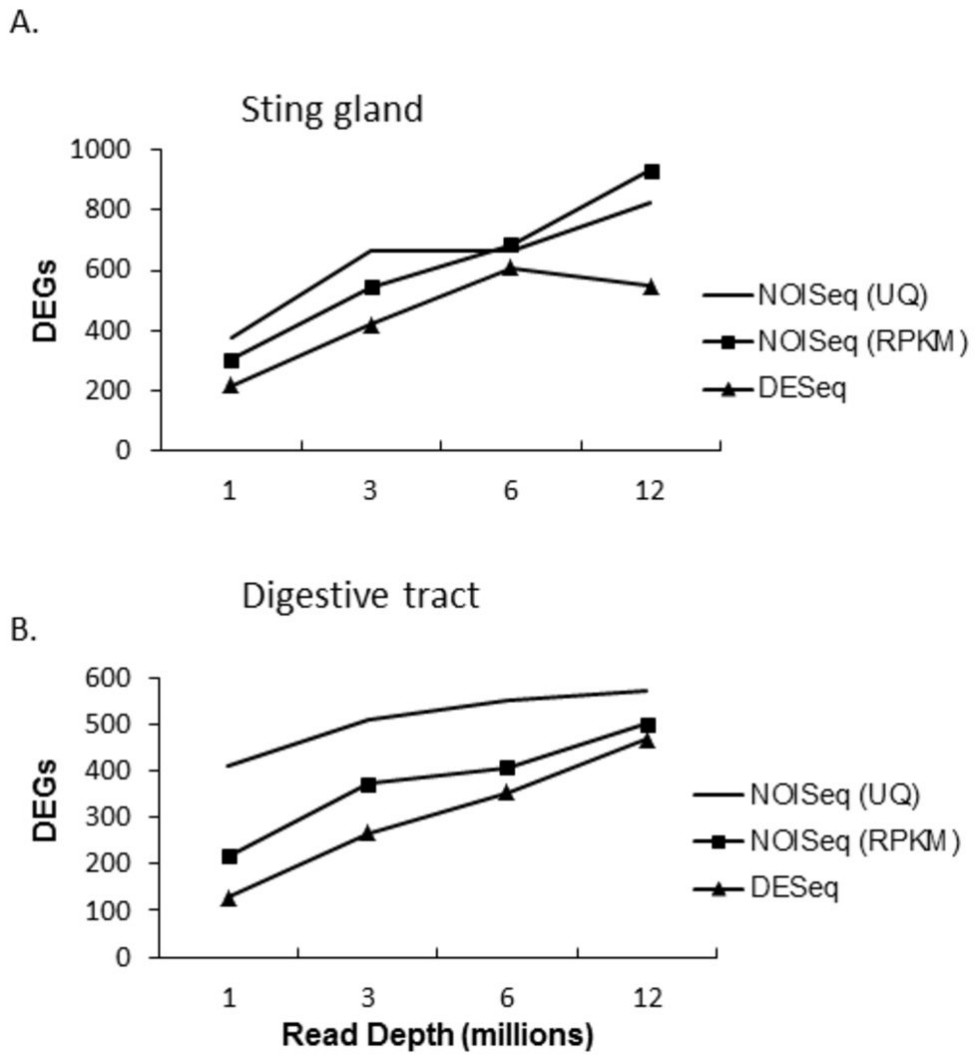
Number of differentially expressed genes found with increasing sequencing depth in comparisons of (A) the sting gland between nurses and foragers, and (B) the whole digestive tract between nurses and foragers. Two different normalization techniques were used in the NOISeq package: upper quartile normalization (UQ), and RPKM.

**Figure 2:**
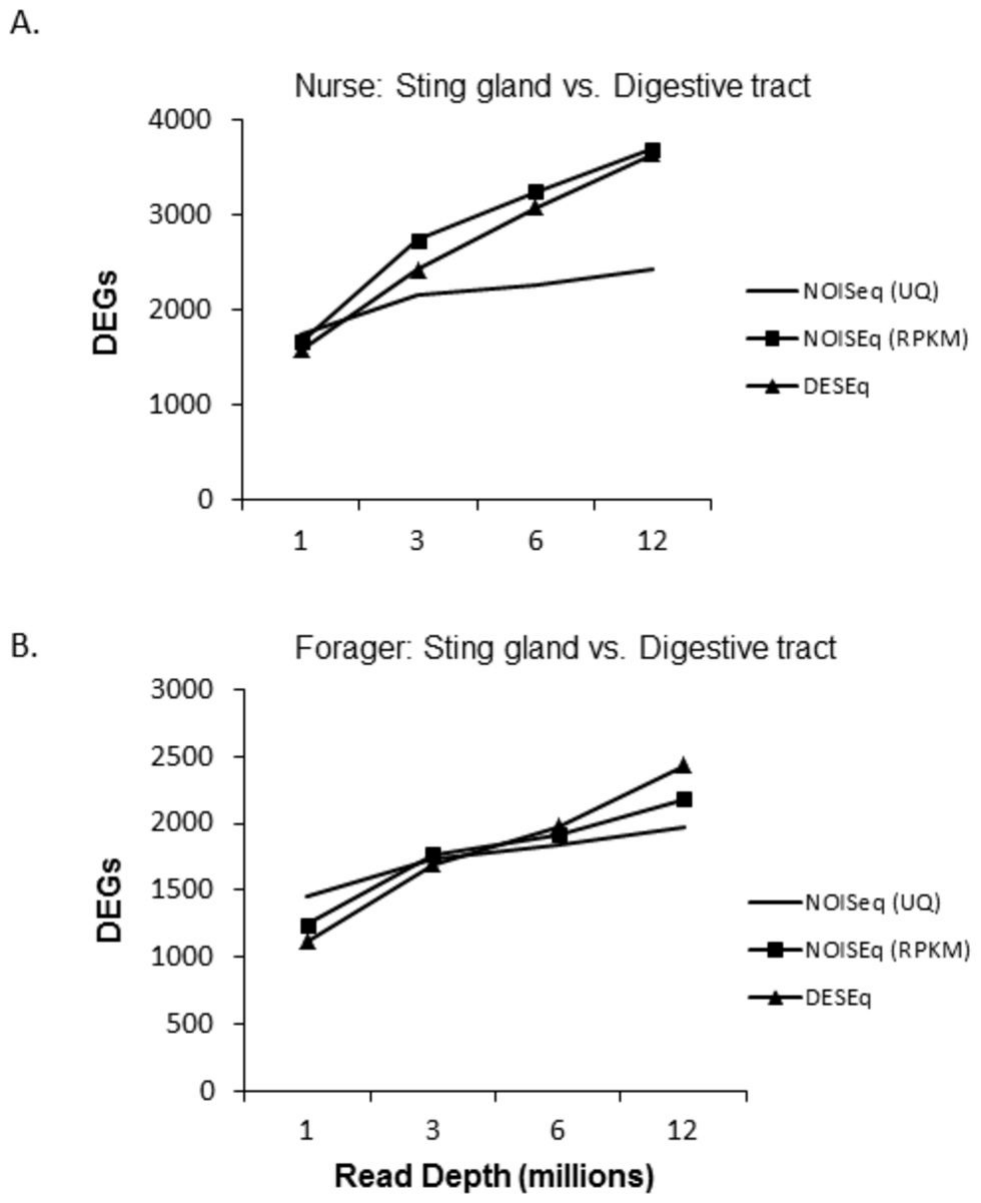
Number of differentially expressed genes found with increasing sequencing depth in comparisons of the sting gland and digestive tract. Nurse sting gland is compared to nurse digestive tract in part A, and forager sting gland is compared to forager digestive tract in part B.

Figures 1 and 2 show the number of differentially expressed genes in each of the two tissues with increasing sequencing depth. Figure 1 compares the same tissues between nurses and foragers (sting gland in nurse versus sting gland in forager and digestive tract in nurse versus digestive tract in forager) and Figure 2 compares tissue types within life history phases (sting gland in nurse versus digestive tract in nurse and sting gland in forager versus digestive tract in forager). Shown are the number of DEGs at 1, 3, 6, and 12 million reads per replicate (2 biological replicates in each group). For both methods, the number of DEGs increased with increasing sequencing depth (expression levels and p values are presented for each differentially expressed gene for each software package in Table S2). For NOISeq this was true with both the RPKM and Upper quartile (UQ) normalizing methods [5,43]. While the number of DEGs was lower for the developmental phase comparisons (nurses versus foragers) relative to the tissue comparisons (sting gland versus digestive tracts), this increasing trend was evident for both. Overall, these data suggest that the sequencing depth here is shallow with respect to the identification of DEGs.

**Figure 3:**
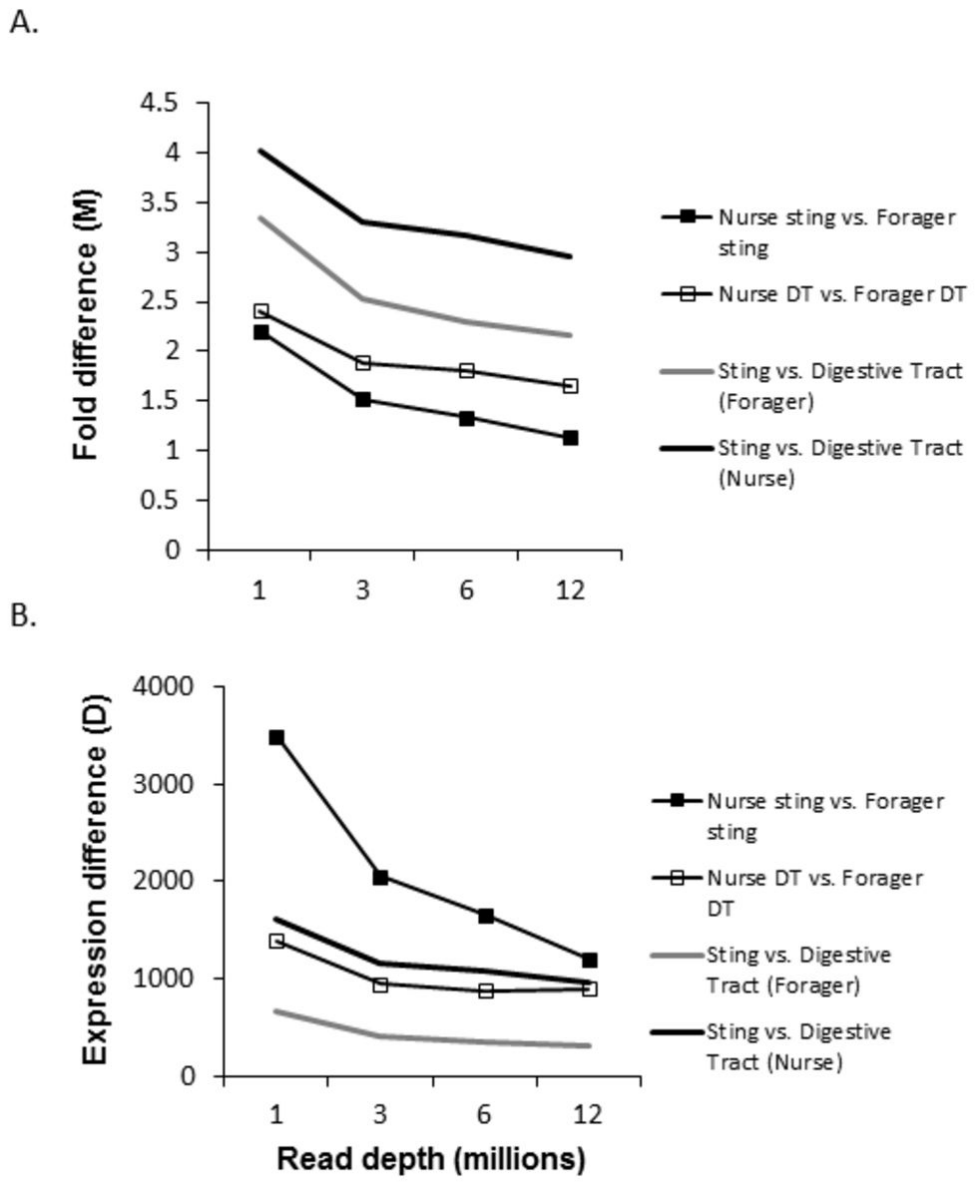
Effect of increasing read depth on the strength of the difference between DEGs measured as M (absolute value of the fold difference in expression) and D (the raw difference in expression).

Figure 3 elaborates on the results of Figures 1 and 2. Here we show that as sequencing depth increases, the magnitude of the differences between DEGs decreases [18]. Differences are measured as the fold difference (M) and the raw difference (D) using the NOISeq data set and following the methods of Tarazona et al [18]. Essentially, M is the absolute value of the log base 2 of the fold difference between expression in the two contexts, while D is the raw difference in expression. The results are consistent both for comparisons of the same tissue between developmental phases (low overall differential expression context) and the comparisons between tissues in the same developmental phase (high overall differential expression context). This finding is important because it suggests that genes with the most robust patterns of differential expression are those found at the lowest sequencing depths. If this is generally true, then it implies that shallow RNA-Seq may be sufficient for the study of such genes.

The result of Figure 3 was shown by Tarazona et al [18] for DESeq and other statistical packages, but not for NOISeq. Tarazona et al argued that NOISeq does not show this pattern of decreasing M and D with increasing sequencing depth because the package is not sensitive to increasing read depth in general. The cause of the difference between our results and theirs is probably due to the fact that we are conducting shallower RNA-Seq then they conducted. Their results are likely for increasing sequencing depth beyond the depth we focus on here. In Figure 3, it is clear that the effect is strongest for low read depth and falls off. It would therefore likely not be seen at higher sequencing depths (it might also not be observed at the same sequencing depth used here, but with more replicates). 

### Focal gene benchmarking

Table 1 shows the eleven focal honey bee genes we used to benchmark how many reads are necessary to identify key genes with tissue-specific functions. These genes are known from experimental data to confer tissue-specific function to the sting gland [29-31]. Most of these genes are venoms, as would be expected, but some are allergens. Table 2 shows that even at low sequencing depth, most of the genes are identified as differentially expressed between the nurse and forager sting gland libraries, using both software packages. With the NOISeq package, all eleven are found using only 3 million reads per replicate. This was true for both the life history comparisons (nurses versus foragers) and the tissue comparisons (sting gland versus digestive tract). The DESeq package did not perform as well, missing a few key genes with low expression levels. Table S3 shows the expression levels for all of these genes at the full 12 million reads depth. In conclusion, it is possible to identify many genes conferring tissue specific functions with shallow RNA-Seq using only two replicates. In this case, we were able to identify all the most important genes conferring the specialized function of this gland at very low depth.

**Table 1:** Key tissue-specific genes in the sting gland.

Gene #	Name
GB10355	melittin precursor
GB11552	venom serine protease 34 precursor Api m 7
GB12546	venom acid phosphatase Acph-1 precursor Api m 3
GB13285	mast cell degranulating peptide preproprotein
GB13351	phospholipase A2 precursor
GB13967	icarapin-like precursor
GB14496	venom dipeptidyl peptidase 4 precursor Api m 5
GB16587	C1q-like venom protein precursor
GB18161	apamin preproprotein
GB18543	hyaluronidase precursor
GB19783	allergen Api m 6 precursor
GB19804	secapin preproprotein

**Table 2:** Number of key sting gland genes (out of 11) found with shallow RNA-Seq.

Sting Gland(Nurse vs. Forager)	NOISeq (RPKM)	NOISeq (UQ)	DESeq
1 million reads	10	10	8
3 million reads	11	10	9
6 million reads	11	10	9
12 million reads	11	10	10
Sting vs. DT (Nurse)	NOISeq (RPKM)	NOISeq (UQ)	DESeq
1 million reads	10	11	9
3 million reads	11	11	11
6 million reads	11	11	11
12 million reads	11	11	11
Sting vs. DT (Forager)	NOISeq (RPKM)	NOISeq (UQ)	DESeq
1 million reads	10	10	7
3 million reads	10	10	9
6 million reads	10	10	9
12 million reads	10	10	9
Total Focal Genes	11		

### Are gland transcriptomes highly specialized?

**Figure 4:**
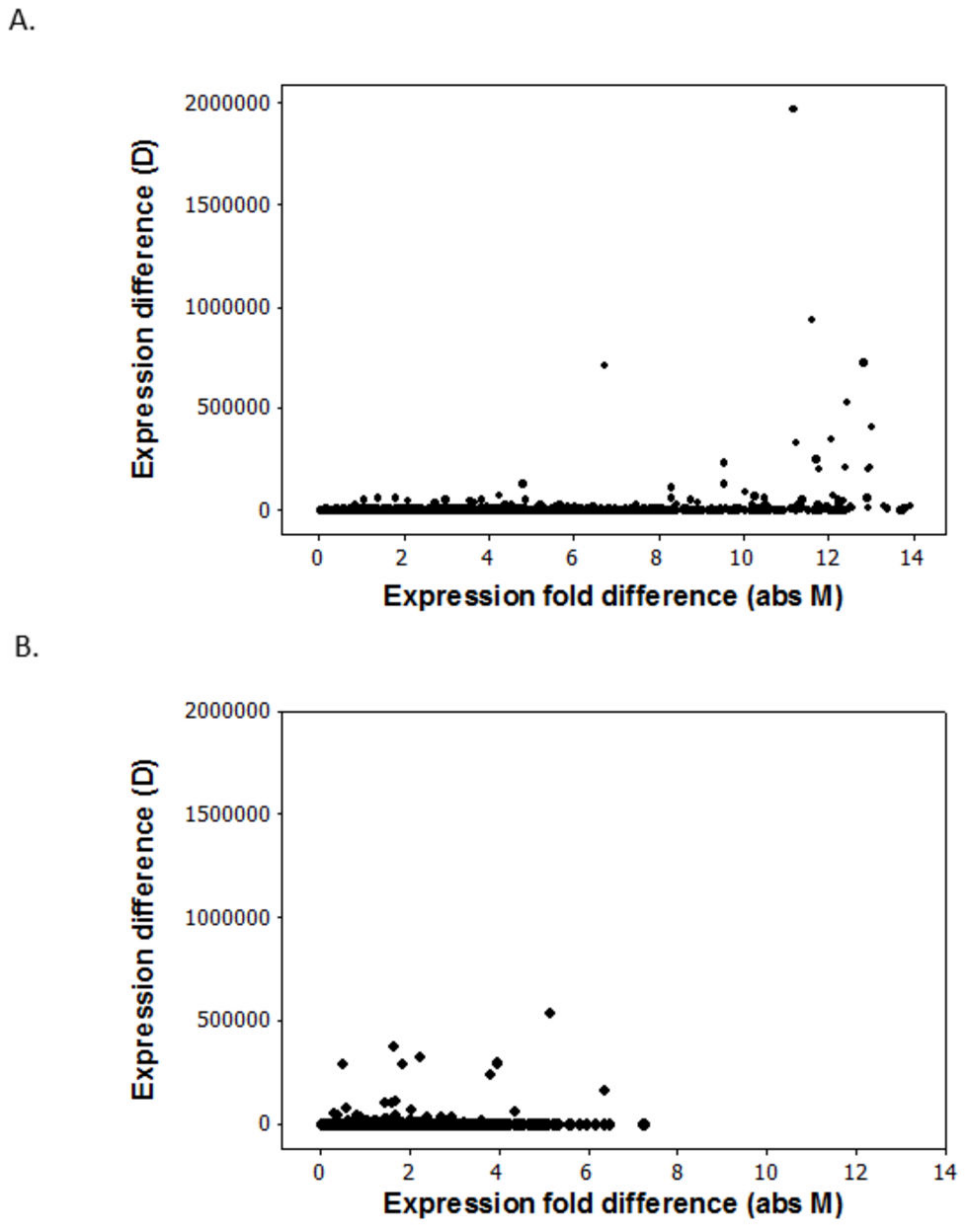
Relationship between fold difference (M) and raw expression difference (D) for all expressed genes in the sting gland in nurses versus the sting gland in foragers (part A) and the digestive tract in nurses versus the digestive tract in foragers (part B).

A criticism of the broad significance of the results of the previous section might be that venoms, and genes important for glands in general, may show very high expression levels, making them easier to identify than genes with functions in other tissues. This would follow from the specialized nature of glandular tissue. Table S3, however, shows that the functionally important genes in the sting gland display a diversity of expression levels. Some of them represent genes with the highest levels of expression, while others have average or low levels. This suggests that the functionally important DEGs that we focus on are not easy to identify merely because they are uniformly highly expressed. However, a transcriptome level comparison of differential expression between the two tissue types used in this study (sting gland and digestive tract) does indicate that glandular tissue is different with respect to the overall pattern of differential expression. Figure 4 shows that the gland tissue contains many more genes with both very high M (fold difference) and D (raw difference) levels than does the digestive tract. This is true for comparisons of the sting gland in nurses versus the sting gland in foragers (part A), and for comparison of the digestive tract in nurses versus the digestive tract in foragers (part B). This result is consistent with the notion that glandular tissue is specialized for producing some transcripts at very high levels. Further, from Table S3 it is evident that the genes with high expression levels, and high M and D values, are also the genes that are easiest to identify with shallow RNA-Seq. In conclusion, based on the sting gland analyses alone, shallow RNA-Seq appears to be quite useful for exploring the specialized functional basis of glandular tissue. However, it is not clear that these results extend to non-glandular tissue. 

### Digestive tract focal gene analysis

Given that the results of the glandular analysis are difficult to extend to other tissue types without further data, we repeated our basic approach with the digestive tract. The digestive tract in honey bees is not as well understood as the sting gland. However, the function of digestive tracts in general is somewhat straightforward to predict, given the clear purpose of this tissue. To explore the utility of shallow RNA-Seq for the study of the digestive tract, we began by annotating with Blast2GO all the DEGs found in the digestive tract [44]. We focused on DEGs between the digestive tract in nurses versus the sting gland in nurses, because this is the context with the largest number of DEGs and hence the most difficult context for identifying key DEGs. Our basic question is whether genes found at low sequencing depth to be DEGs are strongly biased towards being key genes conferring tissue-specific functions. We therefore compared the GO categories of the top 50 DEGs found using NOISeq with all the DEGs found (565 total). 

**Figure 5:**
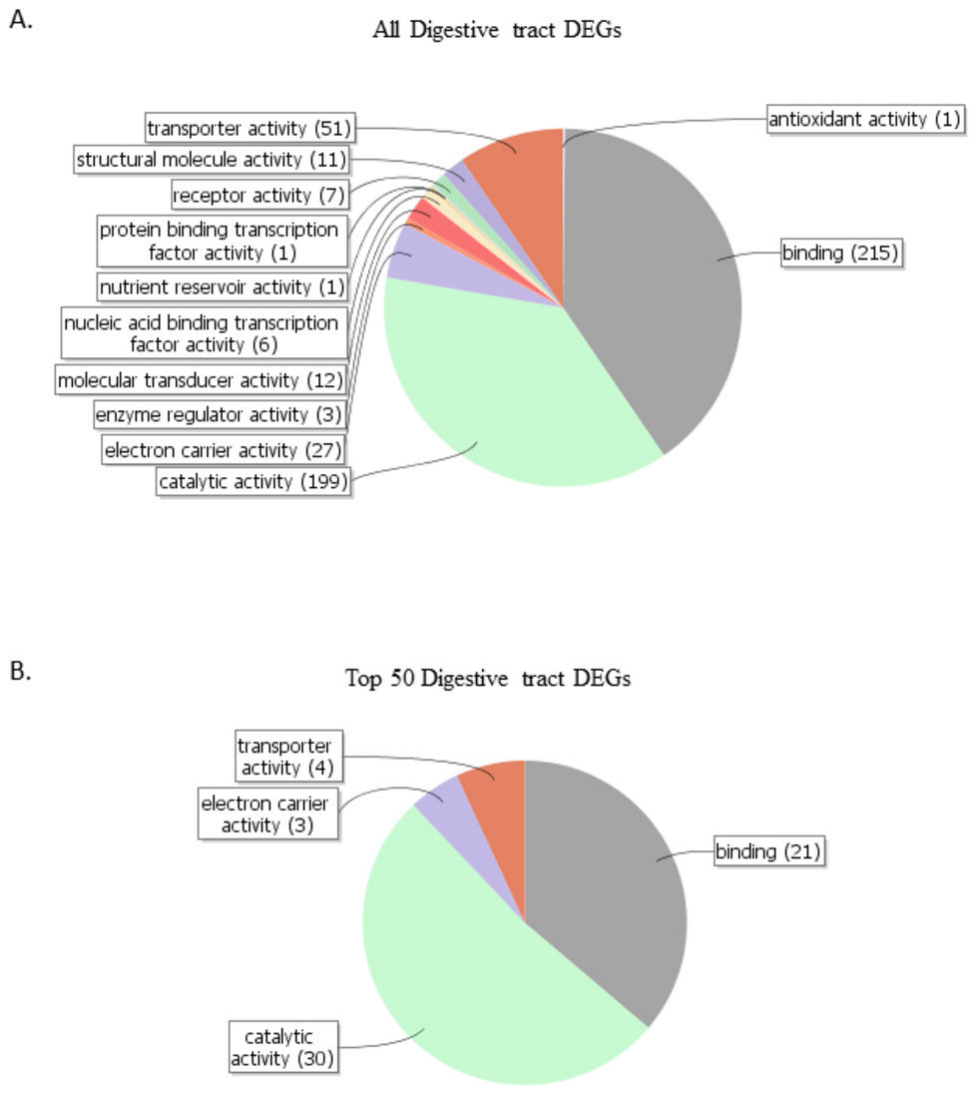
Gene ontology analysis of differentially expressed genes between nurse and forager digestive tracts. Top panel shows the distribution of GO functional categories for all DEGs found (565 in total), while the bottom panel shows the same for the genes with the 50 highest probabilities of being differentially expressed (the 50 genes with the most robust patterns of differential expression).

Figure 5A shows the distribution of GO categories for all the DEGs found to be up-regulated in the digestive tract relative to the sting gland in nurses. There is a strong bias towards genes with digestive functions, but many GO categories are represented. Figure 5B shows the distribution of GO categories for the top 50 genes most likely to be differentially expressed (based on the NOISeq p values). Again, catabolic functions are the dominant category. The large fraction of genes with binding functions is also primarily within the context of digestion (Table S4). Although there are a smaller total number of categories relative to the GO analysis for all the DEGs, the fraction of genes with catabolic functions is nearly the same. Hence, it is not the case that only those genes with the highest probability of being DEGs are strongly biased towards conferring tissue specific functions. In contrast, all the DEGs are strongly biased towards being genes with tissue-specific functions. It may, however, be the case that the genes with the most robust patterns are those digestive genes playing the largest functional role, since these genes show the narrowest pattern of GO categories specific to this tissue’s functional role. 

 To provide further insight into the digestive tract analysis, we carried out qPCR on 10 genes (Figure S1) that were found to be differentially expressed at the 12 million read sequencing depth. For seven of these genes, the results for all 3 biological replicates were in the same direction as the RNA-seq results. The other three genes (GB14596, GB13450 and GB19617) had relatively low expression levels (Table S2). We sought to determine the relationship between the qPCR and RNAseq results as sequencing depth varied (Table S5). We calculated Spearman’s rank correlation coefficient for the fold change at each sequencing depth and the average qPCR fold change across biological replicates. This coefficient increases from 0.82 at the 1 million read depth to 0.84 at the 3 million read depth, but does not increase further at higher depths. Comparable results were seen when the Pearson product-moment correlation coefficient was calculated (Table S5). Since increasing the sequencing depth beyond 3 million reads, for the genes assayed, has little effect on the statistical relationship between the RNAseq and qPCR fold change, it appears that relatively shallow sequencing may be sufficient to capture the robust biological information that can be cross-validated with a different platform using different replicates of the same tissue types.. 

### Transcription factor analyses

Transcription factors, and other regulatory genes, are thought to show lower expression levels than other classes of genes [26-28]. This is because the action of a regulatory protein, to modify expression of other genes by interacting with DNA and or proteins involved with transcription, may not require as many copies of the protein as is necessary for an enzyme or a protein destined for export. Given that our focal gene analysis was with secreted proteins and enzymes, a necessary counterpoint to this analysis is one that focuses on genes, such as TFs, that show lower levels of expression.

 A conservative list of honey bee TFs was generated by running BLAST [45] with all *Drosophila* genes with the GO term “sequence-specific DNA binding transcription factor activity” against all honey bee genes in the official gene set. Bee genes with a significant blast hit (e < 10^-20^ ) to one of the fly TFs were kept for further analysis. Blast2GO was then used to annotate these genes. Genes with a DNA binding functional domain in the context of regulating transcription were identified. Overall 462 TFs were computationally identified in the honey bee official gene set (Table S6). While this is not a comprehensive list of honey bee TFs, it is a broad list and should contain TFs involved in most biological functions.

**Figure 6:**
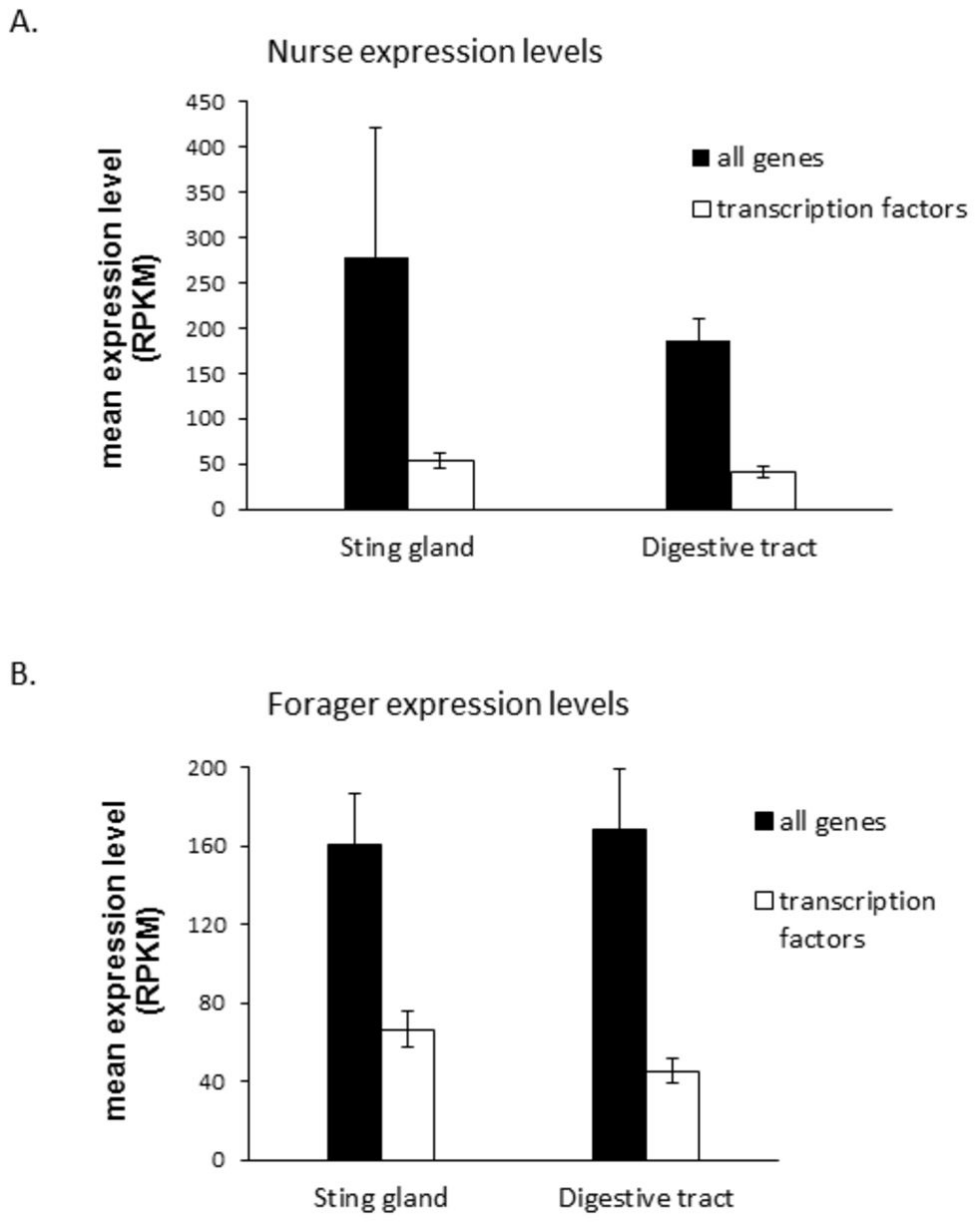
Mean expression levels for all genes and all expressed transcription factors in the sting gland and digestive tract of nurses and foragers. Values are from the 12 million read depth NOISeq analysis.

In total, 257 (55.6%) of the identified TFs (along with a small number of other classes of regulatory genes) were found to be expressed in at least one of the two tissues (Table S7). Figure 6 shows that the assumption that TFs are expressed at relatively low rates is correct for both tissues. TF expression rates are much lower than the average rate of expression for all genes (Tables S7; S8). All comparisons show significantly lower rates of expression for TFs, except for the comparison of the nurse sting gland TFs against all the genes expressed in the nurse sting gland. This result was caused by the enormously high expression level of melittin in this tissue causing a very high standard deviation. If this outlier is removed, this test is also significant. 

**Figure 7:**
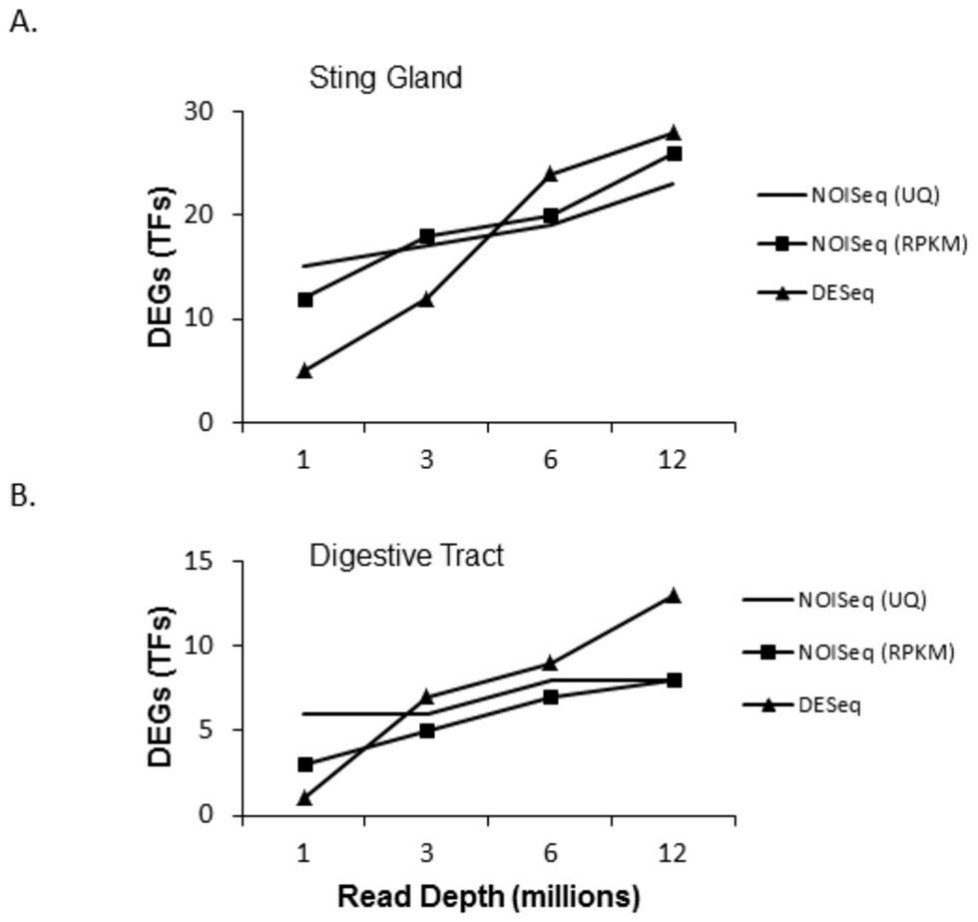
Number of differentially expressed transcription factors found with increasing sequencing depth in the developmental phase comparisons between nurses and foragers.

**Figure 8:**
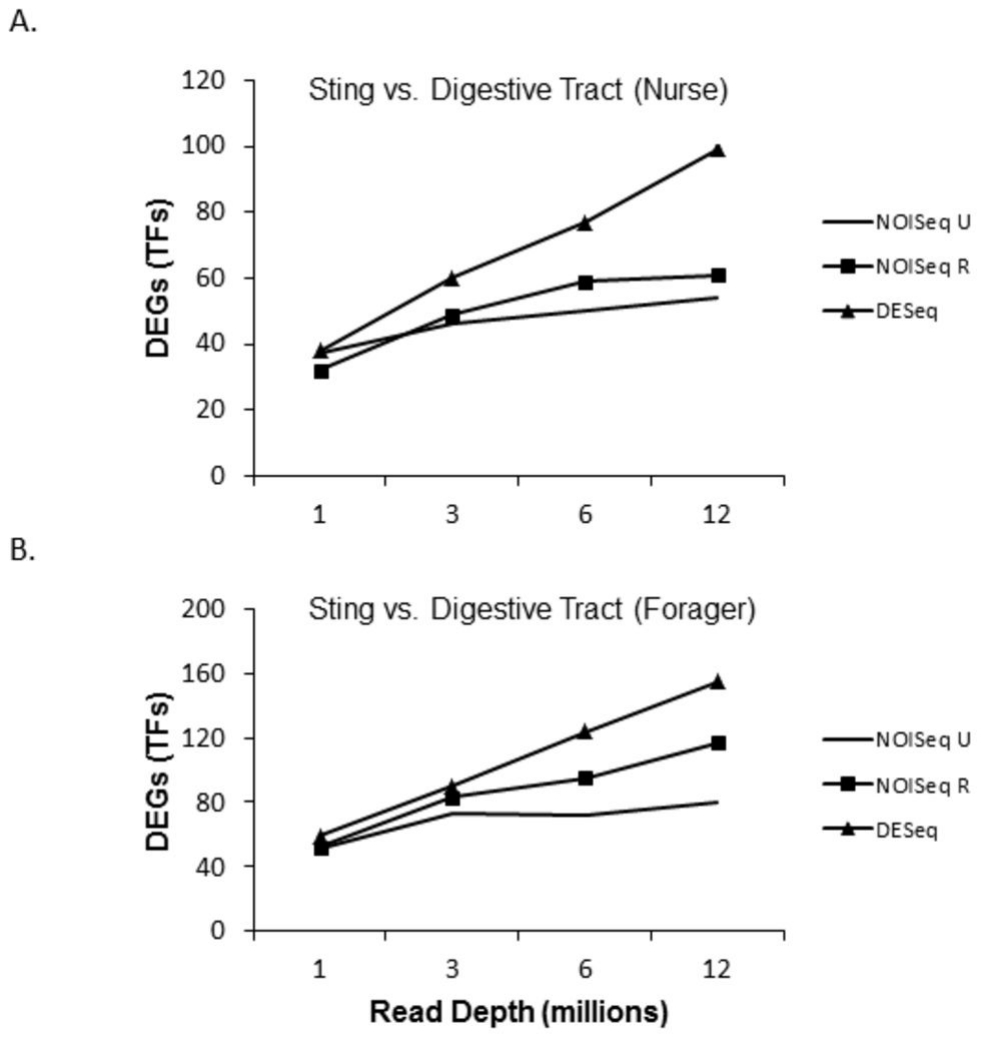
Number of differentially expressed transcription factors found with increasing sequencing depth in the sting gland versus digestive tract comparisons.

**Figure 9:**
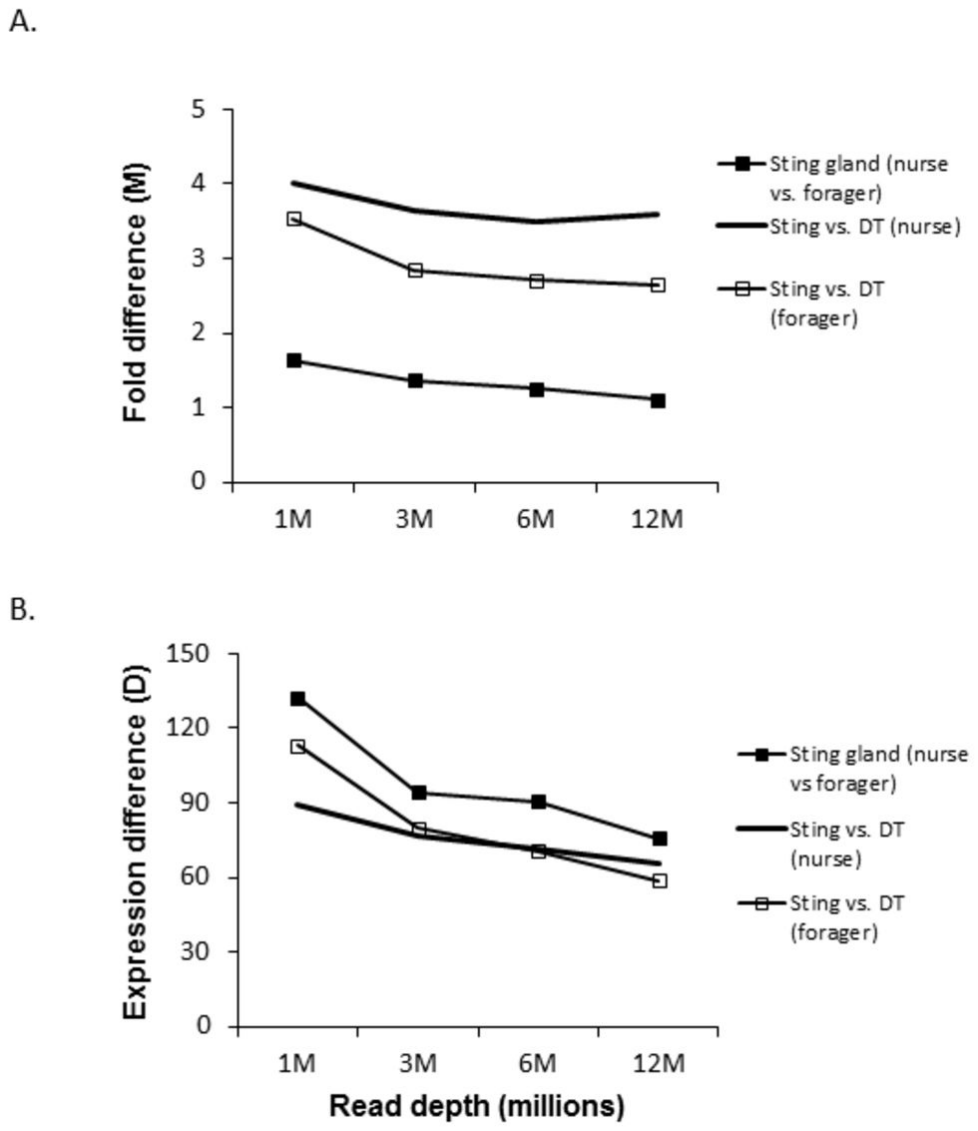
Effect of increasing read depth on the strength of the difference between differentially expressed transcription factors.

Figures 7 and 8 repeat for TFs alone the analyses on the number of DEGs found with increasing sequencing depth. For the comparisons between developmental phases, low, but increasing numbers, of differentially expressed TFs were found with increasing sequencing depth. For the comparisons of the sting gland versus the digestive tract, larger numbers of TFs were found to be differentially expressed with the same increasing pattern. Figure 9 shows that the same basic pattern of decreasing fold and expression difference in DEGs with increasing sequencing depth also holds for TFs. This analysis was limited to the sting gland comparison between nurses and foragers, and the sting gland versus digestive tract comparisons for both life history phases, because these data sets had sufficient sample sizes (number of differentially expressed TFs).

 In total, 152 differentially expressed TFs were discovered using shallow RNA-Seq in this study. Although our bioinformatics pipeline for identifying TFs led to a small number of some other classes of regulatory gene also being identified, the overwhelming majority of genes in Table S7 are clearly TFs. However, it is difficult to know how many false negatives there are (TFs not in Table S7 that should be there). The average expression level for TFs is ≈ 40-70 (RPKM) in this study (Figure 6), and based on Figure 9, the average expression difference at 12 million reads is 70-90 (RPKM). Hence, it appears that only TFs with above average expression levels were found to be differentially expressed in this study. Further, given that the fold difference (M) does not fall much with increasing read depth, it is possible that many more transcription factors show differential expression (albeit with a somewhat lower fold difference) between the tissues we explored. However, it is also possible that differentially expressed TFs are characterized by the largest M values, as clear and strong differences between regulatory proteins may be critical for the control of cell activity. If this is true, then we may have found most of the TFs important for the regulatory control of these two tissues in the adult honey bee. Future work will have to address these issues.

In general, it is difficult to benchmark our TF results since few TFs have been experimentally shown to be important in adult honey bees. This is because little work has been conducted on the topic. Krüppel homolog 1, however, has been shown to be important and differentially expressed between nurses and foragers [46]. This TF shows average expression levels, for a TF, in our data set, and is found to be differentially expressed in the sting gland and digestive tract between nurses and foragers. It is also differentially expressed between forager sting glands and digestive tracts, but not between nurse sting glands and digestive tracts. We were therefore able to recover existing work on *Apis* TF differential expression. In conclusion, although deeper sequencing may be necessary to exhaustively document differential expression of TFs, our work suggests that shallow RNA-Seq may be useful for studying a large number of TFs important for controlling gene expression in adult insects. 

### Identifying key differentially expressed genes

A common problem in RNA-Seq, and microarray studies, is that thousands of DEGs are found and it is not clear which ones are good candidates for further analysis. In our study, at 1 million reads sequencing depth the majority of the focal venom genes were found in the comparisons between developmental states. Significantly, at this sequencing depth, only 200 genes were identified as differentially expressed. Hence, with shallow RNA-Seq, a frequent problem of microarrays and RNA-Seq, huge numbers of candidate DEGs to choose from, may not always be an issue. The number of DEGs to search through for key functional genes can be quite small if the analysis is done with few reads (and few replicates). Further, as Table S3 shows, the focal genes in our analysis would have been high candidates for key genes even without our experimental knowledge. This is because these genes have high M and D levels. If it turns out to be true in general that genes that confer tissue specificity, and TFs, show such robust expression patterns, then shallow RNA-Seq should prove highly valuable. 

 The experimental system, however, will also have a bearing on the choice of sequencing depth. When species can be bred in captivity with relative ease and tissue samples are plentiful and readily available, as in the case of honeybees, the failure of an experiment does not compromise an entire research program. The researchers in this case might opt to for shallow RNA-seq, conserving financial resources and allowing them to multiplex libraries from more than one project on a single sequencing lane, with the knowledge that additional samples are available that could be sequenced at greater depth should the need arise. On the other hand, in situations involving rare or unique samples, such as forensic medicine, the use of shallow RNA-seq would entail a certain risk. In such cases, where the need to make maximum the use of limited material is critical, deeper sequencing would be more prudent.

### Applications to other systems

Although our work shows clearly that shallow RNA-Seq can be of utility for studying patterns of gene expression in some invertebrate tissues, future work will have to determine its utility for other systems. For simpler organisms, such as many single-celled organisms, it may be the case that even shallower sequencing may be sufficient for many applications. This is because the genomes and transcriptomes of such organisms are simpler than for organisms like the honey bee. For vertebrate systems, in contrast, it may be the case that deeper sequencing is necessary for many applications. This is because vertebrates have more complex transcriptomes, which may require greater sequencing depth. Greater sequencing depth is also likely necessary for calling differentially expressed alternatively spliced genes. The depth necessary for the study of different classes of functional RNAs is also unresolved.

However, it is also possible that the depth explored here (up to 12 million reads) may be sufficient for many vertebrate applications. This is because calling differentially expressed genes (based on the present study) appears to be more a function of degree of difference between expression levels in terms of ratios of expression rather than raw numerical difference. This is supported by Table S7, which shows that for the transcription factors studied in this work, expression levels were low, but the ratio of expression between one treatment and the other was high. In this context (and when variance between biological replicates is low), current RNA-Seq software packages are able to identify differentially expressed genes. Hence, complexity of the transcriptome may not interfere with calling differentially expressed genes at relatively shallow depth as long as the ratio of expression for a given gene between treatments is high (even if raw expression levels are low due to the shallow depth). This is speculative, of course, and future work on a variety of systems will be necessary to resolve these issues.

### Utility of Shallow RNA-Seq

Functional genomics is a rapidly evolving field in which the nature of the discipline can radically change over the course of just a few years. This is nowhere clearer than with respect to the question of sequencing depth. The earliest machines did not allow for deep sequencing depth, or for the inclusion of many biological replicates, because the number of reads produced per run was low. With machines such as the HiSeq 2500, which produces hundreds of millions of reads per lane, it is now possible to run many replicates and or experiments per lane. In general, the current goal is not to generate as many reads as possible for each replicate (which would be wasteful in many contexts) but rather to correctly identify the number of reads necessary for a particular application (how many reads per replicate and how many replicates are necessary). In this context, the utility of shallow RNA-Seq is clear. Shallow RNA-Seq is not useful for exploratory studies (first studies of systems). Rather, a first RNA-Seq study would do well to test many read depths in order to find the level at which the number of DEGs plateaus for a given class of genes. Once this number is known (and it may be much lower than would have been thought a priori) then future studies can be designed that take this into account. Such studies would not waste sequencing resources by generating orders of magnitude more reads than are necessary. This will also allow for the running of more biological replicates, since each can be at lower depth. In general, for the genes studied here, shallow depth is sufficient for ongoing studies of these systems. There are likely many analogous biological systems and it is these systems for which shallow RNA-Seq should be the favored approach.

## Conclusions

Although RNA-Seq is quickly revolutionizing the study of large scale patterns in gene expression, there is still much work to be done to determine how best to use this technology in model and non-model systems. In this study, we showed that shallow RNA-Seq can be a powerful tool for analyzing differential expression in genes important for conferring tissue-specific functions. We also for the first time document large-scale patterns of differential expression in transcription factors in a social insect, showing that shallow RNA-Seq can be useful for the study of these genes. This work should be of value to researchers designing RNA-Seq studies in many different disciplines, but further work, perhaps using a similar methodology, is necessary. In partiucalr, studies that use experimentally well characterized vertebrate systems to benchmark how to best replicate those results with and RNA-Seq approach (the experimental design used here) would be useful.

## Supporting Information

Figure S1 **Results of the real-time PCR analysis of 10 genes that show differential expression in the digestive tract RNA-Seq analysis.** Each colony is a different biological replicate, and the error bars show the standard error of the mean of the technical replicates.
(PDF)

Table S1 **Primers used in the qPCR analysis.** CG10903 was used as the reference gene.
(DOCX)

Table S2 **Results of RNA-Seq analyses for Sting gland and digestive tract using DESeq and NOISeq.** Differentially expressed genes, expression levels, and p values are included.
(XLSX)

Table S3 **Expression levels for sting gland genes.** Results are based on the NOISeq analysis focused on comparing the sting gland between nurses and foragers (12 million reads in two biological replicates). The M value is the absolute value of M, while the ‘prob’ is the probability of being differentially expressed (0.8 is the cutoff for statistical significance).
(PDF)

Table S4 **Annotations for the top 50 differentially expressed genes (in terms of probability of being DEGs) up-regulated in the digestive tract relative to the sting gland.**
(XLSX)

Table S5 **Comparison of fold change (nurses/foragers) in digestive tract tissue for 10 genes, using real-time quantitative PCR (qPCR) and RNA-seq at different sequencing depths.**
(XLS)

Table S6 **Annotations for honey bee genes identified as having a domain associated with DNA binding in the context of gene regulation.**
(XLSX)

Table S7 **Results of RNA-Seq analyses for transcription factors in the sting gland and digestive tract using DESeq and NOISeq.** Differentially expressed genes, expression levels, and p values are included.
(XLSX)

Table S8 **Comparison of gene expression levels in the entire gene set versus transcription factor expression levels, using t-tests.** SG=sting gland, DT=digestive tract, N=nurses, F=foragers.
(PDF)
