# Supplementary figures and images for: The Utility of Shallow RNA-Seq for Documenting Differential Gene Expression in Genes with High and Low Levels of Expression
Source: PLoS One. 2013 Dec 16;8(12):e84160. doi: 10.1371/journal.pone.0084160 (PMC3865247; doi:10.1371/journal.pone.0084160)

GB16016

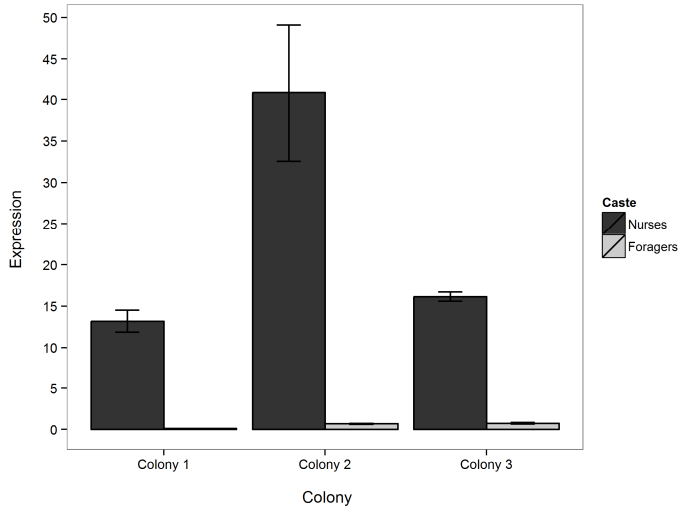

GB16903

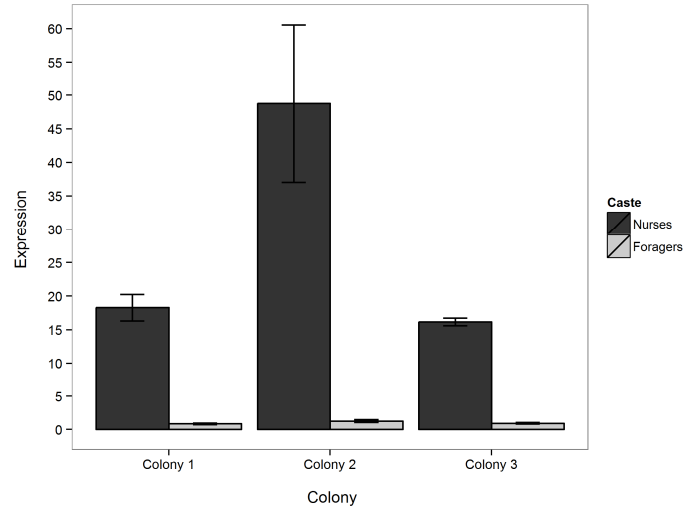

GB13791

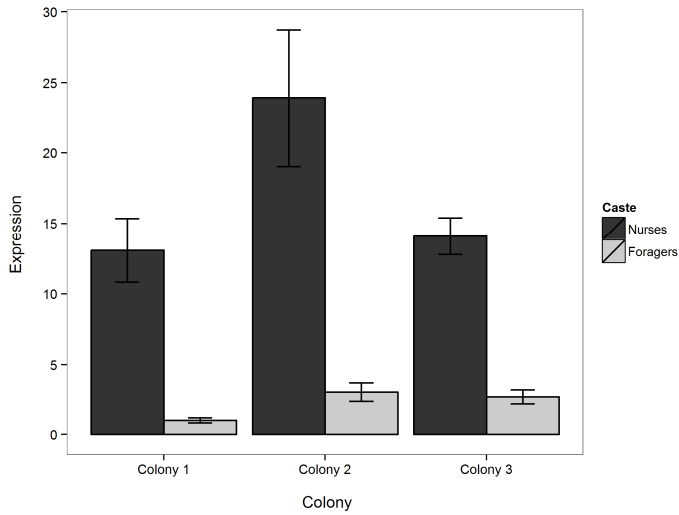

GB13966

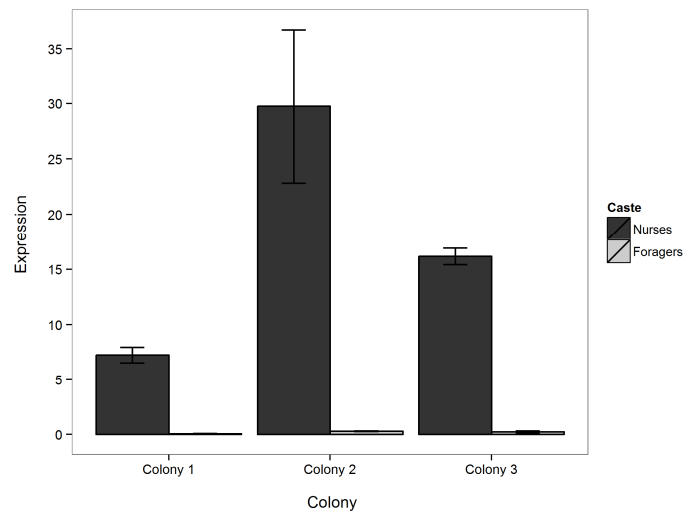

GB14261

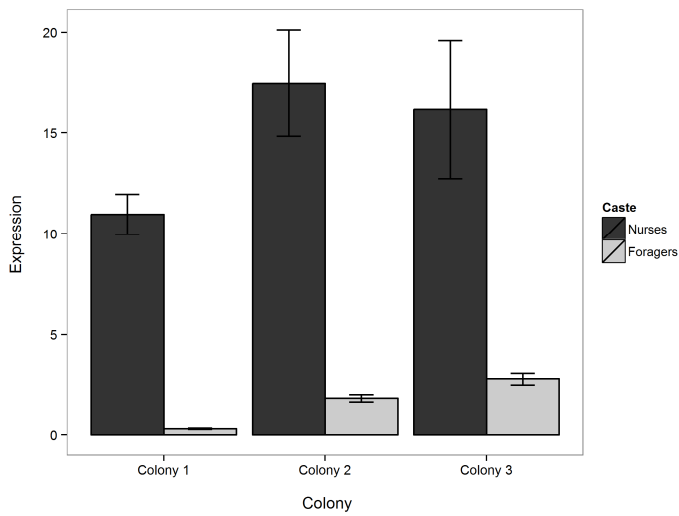

GB14596

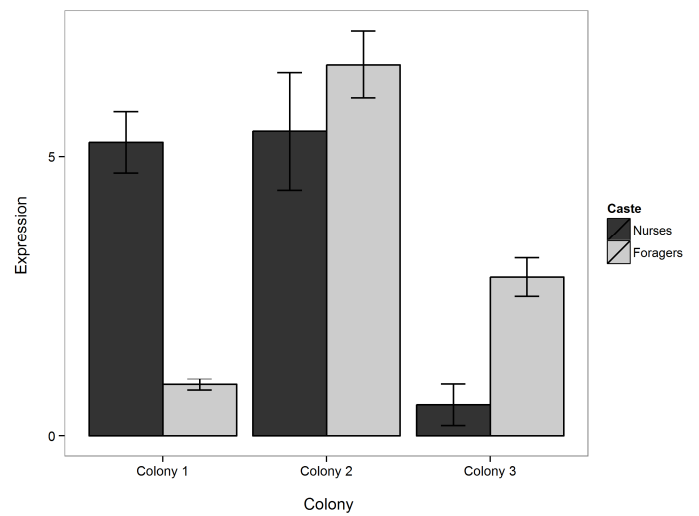

GB18312

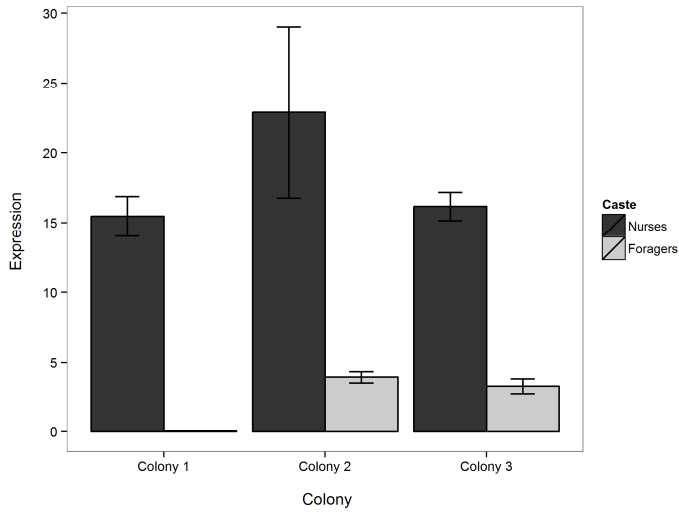

GB18912

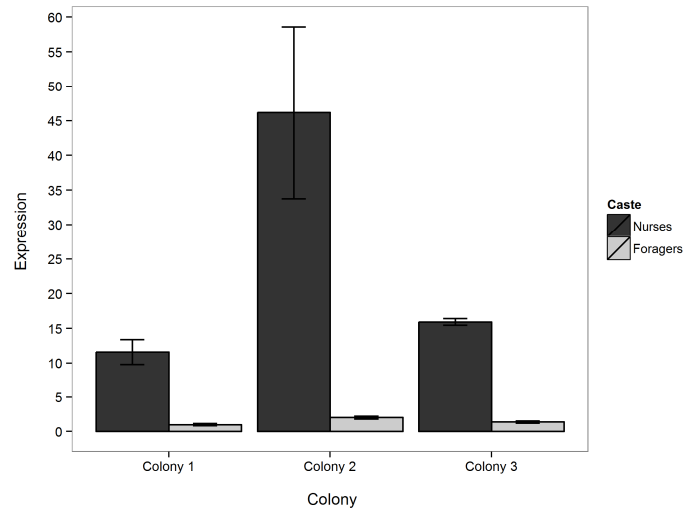

GB13450

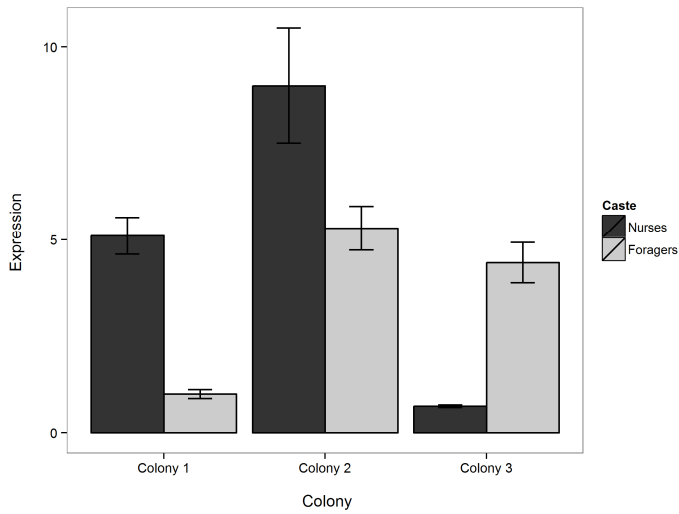

GB19617

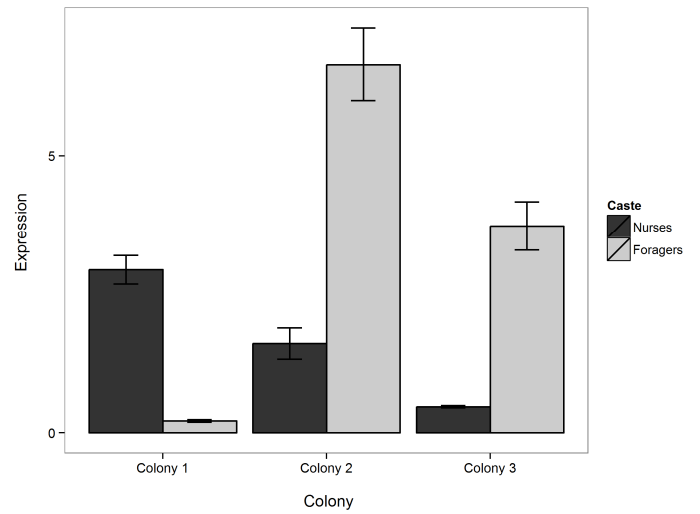

Supplement: Figure S1 — Results of the real-time PCR analysis of 10 genes that show differential expression in the digestive tract RNA-Seq analysis. Each colony is a different biological replicate, and the error bars show the standard error of the mean of the technical replicates. (PDF) [file pone.0084160.s001.pdf]
