# Supplementary material for: The Utility of Shallow RNA-Seq for Documenting Differential Gene Expression in Genes with High and Low Levels of Expression
Source: PLoS One. 2013 Dec 16;8(12):e84160. doi: 10.1371/journal.pone.0084160 (PMC3865247; doi:10.1371/journal.pone.0084160)
Supplement: Table S1 — Primers used in the qPCR analysis. CG10903 was used as the reference gene. (DOCX) [file pone.0084160.s002.docx]

Table S1.

Gene Forward primer Reverse primer

GB16016 CGAGTACATCCTCAGCTTCTCC ACGAATACCACCGTGAGACC

GB13791 GGGCGCACTTAAGCAAGGAG ACCGGCGCATATCATTCTGTC

GB14261 GACCCGTAGTAGATGTTGACC GTAGCTGCTTGTCCACTTTG

GB18912 GCTCTATGGTGCTGTTACTCCTG CCATCACAGCCATTGTTTCC

GB16903 GCCAATGCTGATGTTACGAG GAAAGAGGTTCTGGCTGAGG

GB14596 ACACCTGAAGCTGCATTGAC CAAATCTTCGAGGTGTGGAG

GB13450 GAGCACCTGAACTATTGGTTGG TCCTGGCCATAGTGCTTCTC

GB19617 ATCCAGAACGGGTGTCTCTC CCACCGTTGAATCACTGGAC

GB18312 TTCGTCGACAATCACGACAC GTACCGAATGGATGGGACAG

GB13966 ATCACCGGTGGAGTGAAAGG CCTCGGAGGTCCTCATCTTG

GB10903 GCTGAAATTGCTCATGGTGTG GAGAACGTAACCTTGCACTGG
