# Supplementary material for: The Utility of Shallow RNA-Seq for Documenting Differential Gene Expression in Genes with High and Low Levels of Expression
Source: PLoS One. 2013 Dec 16;8(12):e84160. doi: 10.1371/journal.pone.0084160 (PMC3865247; doi:10.1371/journal.pone.0084160)
Supplement: Table S3 — Expression levels for sting gland genes. Results are based on the NOISeq analysis focused on comparing the sting gland between nurses and foragers (12 million reads in two biological replicates). The M value is the absolute value of M, while the ‘prob’ is the probability of being differentially expressed (0.8 is the cutoff for statistical significance). (PDF) [file pone.0084160.s004.pdf]

Table S3.

	Forager	Nurse	M	D	prob
GB11552	0.58	32.35	5.81	31.77	0.95
GB13351	846	26,053	4.94	25,206	1.00
GB13285	947	24,109	4.67	23,162	1.00
GB19783	127.56	2,109.53	4.05	1,981.97	1.00
GB19804	474.36	4,589.24	3.27	4,114.88	0.99
GB18543	96.28	729.82	2.92	633.54	0.99
GB18161	8,425	52,708	2.65	44,282	0.99
GB14496	17.18	105.03	2.61	87.86	0.97
GB10355	167,302	958,372	2.52	791,069	0.98
GB16587	457.51	1,286.56	1.49	829.05	0.95
GB13967	6,475.49	4,073.50	0.67	2,401.99	0.86
Focal mean	16,834	97,652	3.00	81,255	1.00
Overall mean	611.67	1451.22	1.13	1207.47	0.86
