# Supplementary material for: The Utility of Shallow RNA-Seq for Documenting Differential Gene Expression in Genes with High and Low Levels of Expression
Source: PLoS One. 2013 Dec 16;8(12):e84160. doi: 10.1371/journal.pone.0084160 (PMC3865247; doi:10.1371/journal.pone.0084160)
Supplement: Table S8 — Comparison of gene expression levels in the entire gene set versus transcription factor expression levels, using t-tests. SG=sting gland, DT=digestive tract, N=nurses, F=foragers. (PDF) [file pone.0084160.s009.pdf]

Table S8.

	All genes (n)	Transcription factors (n)	t	p-value
SG N	6692	207	1.56	0.12
SG N minus melittin	6691	207	5.88	<0.001
SG F	7025	231	3.41	<0.001
DT F	5583	153	5.59	<0.001
DT F	6105	186	3.95	<0.001
